# Full Functional Sex Reversal Achieved Through Silencing of *MroDmrt11E* Gene in *Macrobrachium rosenbergii*: Production of All-Male Monosex Freshwater Prawn

**DOI:** 10.3389/fendo.2021.772498

**Published:** 2022-03-17

**Authors:** Hai-Jing Xu, Yi-Lai Chen, Yong-Mei Wang, Jing-Yu Luo, Jian-Wen Li, Shuai-Qi Shen, Jin-Shu Yang, Wen-Ming Ma

**Affiliations:** ^1^ College of Biological and Environmental Sciences, Zhejiang Wanli University, Ningbo, China; ^2^ College of Life Sciences, Zhejiang University, Hangzhou, China

**Keywords:** *MroDmrt11E* gene knockdown, *Macrobrachium rosenbergii*, sex reversal, sexual differentiation, all-male monosex population

## Abstract

The freshwater prawn *Macrobrachium rosenbergii* is one kind of important economic aquaculture species and displays remarkable sexual dimorphism. The molecular mechanism of sexual differentiation in *M. rosenbergii* has been primarily unraveled through the research efforts of the androgenic gland and its related genes. However, the understanding of conserved genes involved in the molecular mechanism underpinning sex determination and sexual differentiation of *M. rosenbergii* is still fragmentary. *MroDmrt11E* is a member of the doublesex and mab-3-related transcription factor (Dmrt) gene family and is prominently expressed in the testis. In the present study, *in vivo* knockdown of *MroDmrt11E* at the postlarva stage in male prawn induced a complete and functional sex reversal and achieved the production of an all-male monosex population. Furthermore, a great deal of new information of upregulated and downregulated transcriptions involved in sexual differentiation of *MroDmrt11E* knockdown was enriched by comparative transcriptomic analysis. The effects of RNAi-mediated gene knockdown of *MroDmrt11E* on the differentially expressed and sex-related candidate genes, such as *transformer, fruitless, feminization, insulin-like androgenic gland* gene, *Dmrt* gene family, were primarily focused on, and their possible molecular regulatory relationships in sexual differentiation were analyzed. Meanwhile, the response of primary Kyoto Encyclopedia of Genes and Genomes (KEGG) biological pathways was investigated to expound the potential roles of *MroDmrt11E* in male sexual differentiation, which provided a deeper understanding of the molecular regulatory network underlying sexual differentiation of *M. rosenbergii*. The finding provided a novel sexual manipulation technique through silencing of *Dmrt* gene family for achieving a complete and functional sex reversal and offered a new insight regarding the mechanism of the Dmrt gene family in the sexual differentiation of crustaceans.

## Highlights

1. *MroDmrt11E* silencing induced a complete and functional sex reversal in male juveniles and was applied in the production of an all-male monosex population
2. The effect of RNAi-mediated gene knockdown of *MroDmrt11E* on sex-related candidate genes and the changes of primary KEGG biological pathways were investigated by comparative transcriptomic analysis

## Introduction

The freshwater prawn *Macrobrachium rosenbergii* is an important economic aquaculture species that is widely distributed in tropical and subtropical regions (1). It displays remarkable sexual dimorphism, which means the male prawn grow faster than females and show larger body sizes in adults (2). Thus, to achieve monosex production of this species, considerable research efforts have been devoted to unraveling the molecular mechanism of sex determination and sexual differentiation of *M. rosenbergii* (2–4).

In crustaceans, the androgenic gland (AG) provided much more information in the history of sexual regulation research. AG is known as a male-specific gland and primarily regulates male sexual differentiation and maintains male characteristics in crustaceans. Simultaneously, several insulin-like androgenic gland peptides (IAGs) have been identified in decapods (3, 5–8). Previous studies have reported that IAG from *M. rosengergii* (Mr-IAG) is proven to participate in male differentiation (3, 9). *Mr-IAG* silencing at postlarva (PL) induced a full and functional sex reversal and achieved the production of an all-male monosex population in *M. rosenbergii* (2, 4, 10). Moreover, long-term knockdown of *Mr-IR*, the receptor for Mr-IAG, also induced sex reversal and yielded neo-females in *M. rosenbergii* (11). For another instance, silencing *Cq-IAG* in *Cherax quadricarinatus* feminized male-related phenotypes (6). Until now, in crustaceans, the central role of IAG in orchestrating male sexual differentiation has been well characterized (10, 12), but the understanding of other conserved genes involved in sex determination and sexual differentiation is still fragmentary. On the other hand, identifying and studying the regulation mechanism of sex-related genes and noncoding RNAs also provide valuable sexual development information of decapods (13). Thus, the first question is whether the crustacean develops a novel sexual differentiation pathway, which connects the AG-specific genes and other sex-related regulatory factors.

Dmrt is a kind of well-conserved protein and is characterized by a DNA-binding region called the DM domain (14, 15). All of the arthropod doublesex (dsx) (16–18), nematode mab-3 (19), and vertebrate dmrt1 (20) are members of Dmrt gene family and involved in sex determination and/or sexual differentiation in bilaterian animals. In *Drosophila*, primary sex determination occurs very early in embryonic development when zygotically transcribed genes located on the X chromosome and the autosomes and activate Sxl in females but fail to activate it in males (21). Dsx is the connecting element and the central nexus of insect sex determination (18). Dmrt1 has appeared in the common vertebrate ancestor and is a regulator of male determination and testicular formation in gonadal cells (20). In mammals, *Dmrt1* activates the male sexual differentiation signaling pathway and promotes testicular growth and development (20). Accumulating evidence from these studies suggested that the sexual differentiation functions of Dmrt gene family were evolutionarily conserved (14, 15).

Recently, numbers of *Dmrt* gene family have been identified from several crustaceans, such as the Chinese mitten crab *Eriocheir sinensis* (22), the water flea *Daphnia* (17), the eastern rock lobster *Sagmariasus verreauxi* (23), the Chinese shrimp *Fenneropenaeus chinensis* (24), the oriental river prawn *Macrobrachium nipponense* (25), and the giant freshwater prawn *M. rosenbergii* (26–28). However, one of the limitations of these studies is that these studies have not explained how the *Dmrt* participates in sex determination and/or sexual differentiation pathway.

In *M. rosenbergii*, several Dmrt gene families have been identified, including *MroDmrt11E, MroDmrt99B, MroiDmrt1a, MroiDmrt1b, MroiDmrt1c, MroiDmrt1d, MroDSX, and Mr-Dsx* (26–28). These *Dmrts* show various time-dependent expression patterns and tissue-specific distributions, which suggested that these *Dmrts* were possibly involved in both somitogenesis and sexual differentiation. Among them, *MroDmrt11E* presents a sexually dimorphic expression pattern, and the transcription is prominent in the testis but lower in the ovary (26, 27). Significantly, *MroDmrt11E* knockdown induced a significant decrease of the *Mr-IAG* transcript (26), suggesting that *MroDmrt11E* possibly plays an upstream role in the “IAG-switch” regulatory signaling and participates in the sexual differentiation by directly or indirectly influencing the expression of *Mr-IAG* (26). However, one question that needs to be addressed is: What is the role of *MroDmrt11E* in male sexual differentiation? There is no direct evidence of animal experiments to explore *MroDmrt11E’s* involvement in sex determination and/or sexual differentiation. In *M. rosenbergii*, the position of *Dmrt* gene family in the sexual differentiation signaling pathways remains largely elusive.

The present study was designed to explore the potential role of *MroDmrt11E* in the male sexual differentiation pathway. In this study, *in vivo* knockdown of *MroDmrt11E* in male PL by RNA interference (RNAi) was implemented to evaluate whether *MroDmrt11E *silencing would induce full functional sex reversal in *M. rosenbergii*. Meanwhile, the response of various related gene transcriptions to *MroDmrt11E* silencing and the effect of RNAi-mediated gene knockdown of *MroDmrt11E* on sex-related gene expression were investigated. The main understanding of this study would offer a novel insight regarding the biological function and mechanism of *Dmrt* gene family in regulating sexual differentiation of crustaceans.

## 2 Materials and Methods

### 2.1 Animals

*M. rosenbergii* PL of the same brood was collected from Yonggang commercial farm in Ningbo, Zhejiang, China, and acclimated in laboratory tanks 1 week before injection. The prawn at the PL15–30 stage with 1.0–1.5-cm body length (BL) were selected for the treatment. The BL of *M. rosenbergii* was measured as a straight line from the base of the eyestalk to the end of the telson. The injected shrimps were reared in separated tanks (100 L) under a flow-through system. The temperature was maintained at 27°C ± 2°C, and the photoperiod was 14:10 (light:dark per day). Prawn were fed with artificial food twice daily.

### 2.2 *In Vivo* Knockdown of *MroDmrt11E* by RNAi in Postlarvae

For RNAi experiments, the dsRNAs of *MroDmrt11E* and Green fluorescent protein (*GFP*) were produced and purified as described (26). Here, 5 μg dsRNA of *MroDmrt11E* in 0.9% (w/v) physiological saline was injected into each shrimp (N = 200) through the arthrodial membrane at the base of the fifth pereiopods using a microinjection needle. The control group (N = 200) received an equal amount of *GFP* dsRNA injection. The injection was performed monthly during the animal experiment, and three injections for 90 days were performed for each prawn.

To evaluate the RNAi efficiency, the male reproductive system of the RNAi group (N = 5) was dissected at the end of the animal experiment. The interference efficiency of *MroDmrt11E* silencing was detected by both real-time fluorescence quantitative PCR (qPCR) and semiquantitative-PCR. On one hand, SYBR Green RT PCR assay was carried out in a CFX384 quantitative PCR Detection System (Bio-Rad, USA) for qPCR analysis. Here, *18S* (GenBank accession no. DQ642856.1) was used as an internal reference to adjust the number of cDNA templates. Mro18S-qF 5′-GAGAAACGGCTACCACATCCAA-3′ and Mro18S-qR 5′-GTGCTCATTCCAATTACGCAGACT-3′ were designed to generate a 125-bp fragment of *Mro18S*. And *MroDmrt11E*-qF 5′-CGCATCCCACCCTACTTGA-3′ and *MroDmrt11E*-qR 5′-GGCTTCCCTCTGCATCATGA-3′ were designed to generate an 89-bp fragment of *MroDmrt11E*. A total volume of 20 µl mixture [10 µl of 2× SYBR Master Mix (Applied Biosystems, USA), 1 µl of cDNA mix, 0.5 µl of each primer (10 µM), and 8 µl of sterile distilled H_2_O] was used for qPCR analysis. And the program of qPCR was 95°C for 1 min, followed by 40 cycles of 95°C for 15 s and 63°C for 25 s. Three replicates for each sample were performed. The relative expression level was calculated using the 2^-ΔΔCt^ method. The data obtained from qPCR analysis were analyzed for statistical significance using GraphPad Instat (GraphPad Software Inc.).

On the other hand, one pair of *18S* primers (Mro18S-F 5′-GGTAGTGACGAAAAATAACAAT-3′ and Mro18S-R 5′-CCCACCCCAGTCCGGAACTGA-3′) and another pair of *MroDmrt11E* primers, *MroDmrt11E* (*MroDmrt11E*-F 5′-CACTCCTCCAGTTGGTTGT-3′ and *MroDmrt11E*-R 5′-GCTGATGGGTGTCCTTGT-3′), were used for semiquantitative PCR analysis. The PCR products were assessed by electrophoresis on 1.2% agarose gel. Three replicates for each sample were also performed. Relative abundances were expressed as the ratio of *MroDmrt11E* transcript levels to those of *18S* rRNA. The peak value of the GFP group was set to 100, and the rest of the value was normalized. The data obtained from PCR analysis were analyzed for statistical significance using GraphPad Instat (GraphPad Software Inc.).

### 2.3 The Effects of *MroDmrt11E* Silencing on the Induction of Sex Reversal

#### 2.3.1 Identification of Sex Reversal by *MroDmrt11E* Silencing

In the freshwater prawn *M. rosenbergii*, the manipulation of the insulin-like androgenic gland hormone (*Mr-IAG*) silencing at the PL period obtained a full and functional sex reversal, leading to the production of an all-male monosex population (2). Thus, similar gene silencing is attempted to expound whether *MroDmrt11E* participates in male sexual differentiation in *M. rosenbergii*.

In the knockdown of *MroDmrt11E*, the male/female sex of the silenced individual was inspected and identified by both external appearance observation (1) and genetic molecular marker methods (1, 29). Since gross external signs of first sexual characteristic, such as genital pores and male appendages, are not evident at the PL stage, the gene silencing experiment lasted 3 months until clear sexual differentiation with 4.0–5.0 cm BL of the prawn (1). At the end of the RNAi experiment, male prawn were distinguished from females with the structure of male genital pores located at the coxopodite of the fifth pereiopods. Then, the muscle genomic DNA of the individuals was extracted respectively and used as the template for PCR amplification to evaluate the genetic sex as described (29). Subsequently, the sex reversal genetic male prawn induced by *MroDmrt11E* silencing were discriminated then kept for further analysis.

#### 2.3.2 Confirmation of a Complete and Functional Sex Reversal and the Breeding of Monosex Progeny

The sex reversal male prawn, known as neo-females (N = 14), were then selected and cultured in the prawn hatchery in Ningbo, Zhejiang, China. All of the external appearance, the development and maturation of the ovary, the reproductive behavior, and the copulation process of the neo-females were observed and compared with those of normal females during the whole period of culture. These neo-females were then mated with normal males and spawned, and the embryos were incubated in their brood chambers. Furthermore, the various characteristic parameters of embryonic development, hatching, and metamorphosis of the progeny of neo-females were surveyed in parallel with that of the control progeny.

In addition, 10 embryos of egg-nauplius or egg-zoea period from each progeny were sampled, and the genomic DNA of each embryo was used for genetic sexual identification by amplifying the genetic sex marker. In general, the progeny of the neo-females was expected to be males, known as an all-male monosex population, whereas the progeny of normal females was composed of both males and females. Moreover, with regard to the growth processes of all-male monosex prawn, all of the embryonic development, zoea larvae metamorphosis, juvenile growth, and adult culture were monitored through the whole culture period of 3–6 months in *M. rosenbergii*.

### 2.4 Comparative Transcriptomic Analysis of *MroDmrt11E* Knockdown

#### 2.4.1 RNA Isolation for Transcriptomic Sequencing

To evaluate the potential role of *MroDmrt11E* in the sexual differentiation pathway, the response of various related gene transcriptions and the expression of sex-related genes were investigated by comparative transcriptomic after *MroDmrt11E* gene knockdown in *M. rosenbergii*.

The individual of *MroDmrt11E* silencing showed clear sexual differentiation with 4.0–5.0-cm BL of the prawn at the end of the 3-month experiment. The male reproductive system (testis, sperm duct, and terminal ampullae) and the coxopodite between the third and fifth pereiopods from males were dissected from *MroDmrt11E*-silenced individuals (N = 10), and the organs were mixed together to provide sufficient RNA for the transcriptomic sequencing. Total RNA was extracted using the column TRIzol total RNA isolation kit (Sangon) following the manufacturer’s protocol. The OD260/280 should range from 1.8 to 2.0 to ensure the purity of the RNA sample.

#### 2.4.2 Identification and Classification of Differentially Expressed Unigenes

The transcriptome was sequenced using the Illumina HiSeq. The raw reads were cleaned by removing adaptor sequences, empty reads, and low-quality sequences. The clean reads were assembled into non-redundant (Nr) transcripts. The resulting unigene sequences were then annotated using homology search (BLASTX) with an E-value cutoff of 10^-5^ against an NCBI Nr database, Swissprot, Cluster of Orthologous Groups database (COG), and Kyoto Encyclopedia of Genes and Genomes (KEGG) database. The coding sequence and the direction of the annotated unigenes were determined based on the BLAST results from the four above mentioned databases. For the differential expression analysis, the transcript expression level of the unigenes was measured using the FPKM method (fragments per kilobase per million fragments). Genes were considered differentially expressed in the given library when the p-value was less than 0.05 and a greater than 2-fold change (with the absolute value of log_2_ fold change more than 1).

Furthermore, the effect of RNAi-mediated gene knockdown of *MroDmrt11E* on the expression of sex-related genes was also investigated. The differentially expressed sexual candidate genes were considered when the p-value was less than 0.05 and a greater than 1-fold change (with the absolute value of log_2_ fold change more than 0.5) in comparative transcriptomic analysis. According to these differentially expressed transcripts involved in sexual differentiation or cell proliferation signal, the putative intuitive cascade regulation axis or networks of pathways were illustrated.

## 3 Results

### 3.1 *In Vivo* Knockdown of *MroDmrt11E* by RNAi in *M. rosenbergii*

**Figure 1:**
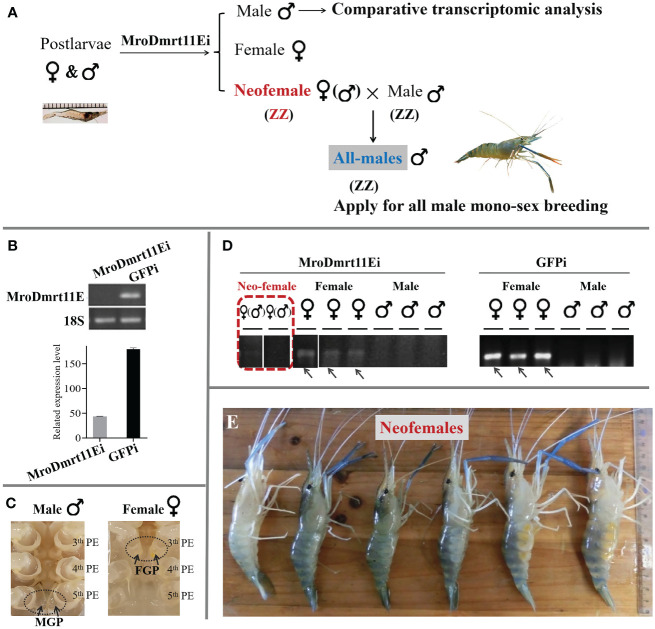
*In vivo* knockdown of *MroDmrt11E* by RNAi in postlarvae. **(A)** The diagrammatic sketch of *in vivo* knockdown of *MroDmrt11E* by RNAi in postlarvae. *MroDmrt11E* dsRNA injection was performed in postlarvae, and the gene silencing lasted for 3 months. **(B)** Detection of the relative *MroDmrt11E* mRNA level in RNAi induced and the control group. **(C)** The sex identification of differentiated male and female prawn by the appearance of sexual characteristics in *M. rosenbergii.* There was a pair of male genital pores located at the coxopodite of the fifth pereiopods in males, but a pair of female genital pores located at the coxopodite of the third pereiopods in females as well as neo-females. MGP, male genital pores; PE, pereiopods. **(D)** The gender of individuals was identified using sex molecular markers in both *MroDmrt11E* RNAi and the control group. The female-specific sex band indicates female genotype. The female-specific sex bands are indicated by the gray arrows in females. The representative neo-female prawn are shown in a red broken line frame. **(E)** The neo-female prawn with female appearance features. The *MroDmrt11E*-silenced genetic males (neo-females) were raised to maturity and induced appearance features of females. Some neo-females spawned and incubated the eggs/embryos in their brood chambers, located ventrally on their abdomens.

The brief strategy of the present study was illustrated in **Figure 1A**. The neo-female prawn was expected to be induced by *MroDmrt11E* silencing and applied for the breeding of the all-male population. The comparative transcriptomic analysis of the *MroDmrt11E* knockdown male reproductive system was implemented to enrich differently expressed genes.

After *in vivo* knockdown of *MroDmrt11E* gene performed in *M. rosenbergii* PL, during the whole RNAi experiment, the mRNA expression level of *MroDmrt11E* was dramatically decreased to a low level compared with control samples (**Figure 1B**). This result indicated that a long-term efficiency of *MroDmrt11E* silencing was successfully maintained. Secondly, the male/female sexes of the *MroDmrt11E* RNAi and control group were identified by both external appearance features (a pair of genital pores) (**Figure 1C**) and the genetic sex molecular marker method (**Figure 1D**). In detail, all female controls in the *GFP*-silenced group and confirmations in the *MroDmrt11E*-silenced group were as expected, revealing the female-specific sex band (**Figure 1D**). Comparatively, although some *MroDmrt11E*-silenced individuals morphologically resembled the control females, without male genital pores at the fifth pereiopods (as evidence of appearance feature in the control male), they lacked female-specific sex bands (as evidence of genetic sex in the control female), therefore proving genetic male (**Figure 1D**). It was clear evidence for sex reversal that *MroDmrt11E* silencing in the PL males successfully induced the full functional sex reversal individuals, also known as neo-females. Moreover, these neo-females were carefully raised to maturity, with induced female appearance features and reproductive system (**Figure 1E**).

### 3.2 Confirmation of a Complete and Functional Sex Reversal and the Breeding of Monosex Progeny

**Figure 2:**
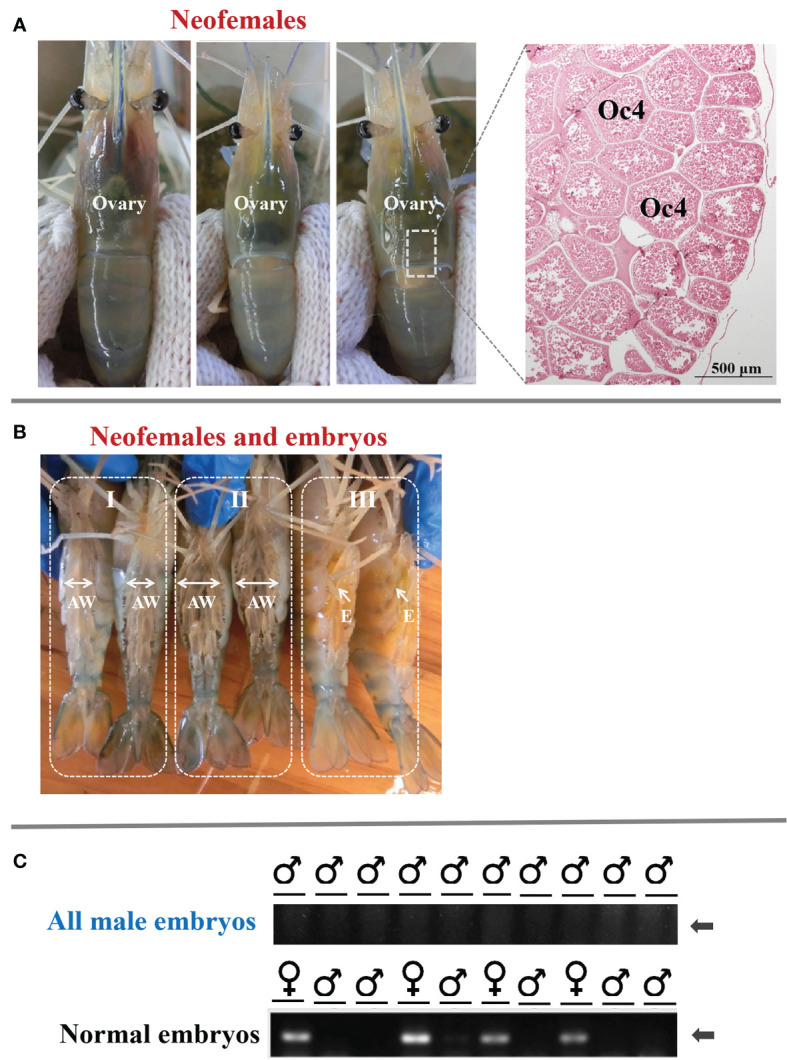
The ovarian development of full-functional sex reversal prawn and the breeding of all-male progeny by *MroDmrt11E*-silenced prawn (neo-females). **(A)** The ovarian development and maturation of neo-females. The inset photograph indicated the histology of the mature ovary of neo-females. The ovary was full of mature oocytes (Oc4) that were intensely eosinophilic (reddish orange). **(B)** Ventral view of *MroDmrt11E*-silenced prawn (neo-females) incubating embryos in their brood chamber. The mature neo-females (B-I) spawned and incubated the embryos in their brood chambers, located ventrally on their enlarged abdomens (B-II, B-III). The color of embryos presented gradual changes from yellow to orange (B-III) then to light-gray color (B-II) during embryonic development. AW, abdomen; E, embryos. **(C)** Genomic validation of representative embryos from each progeny of neo-females. The first row of PCR products was generated from neo-female embryos, and the second row was from normal females. The presented specific band in gel electrophoresis implied the female embryo.

To investigate the potential role of *MroDmrt11E* in gonad development, the *MroDmrt11E*-silenced genetic males (neo-females) were raised into adults. The result showed that these neo-females were characterized by the disappearance of male characteristics (such as male genital pore and male reproductive system) and the formation and development of female features (female reproductive system). Moreover, the mature neo-females developed normal-appearing ovaries as compared with normal females. As to the ovarian development of *M. rosenbergii* neo-females, it was closely related to the individual development process by significant changes in color and ovarian volume. With the gradual development and maturation of the ovary, the full and inflated orange-yellow ovary was displayed throughout the carapace or extended to the first ventral segment (**Figure 2A**).

On the other hand, the breeding of neo-females and their offspring culture was also implemented. The neo-females had successful mating and showed normal mating behavior when they mated with normal males (data are not presented). These neo-females then spawned and incubated the embryos in their brood chambers, located ventrally on the abdomens (**Figure 2B**). The appearance color of embryos presented gradual changes from yellow to orange, then to light-gray color during embryonic development in female (enlarged) abdomen (**Figure 2B**).

Furthermore, to confirm the sex ratio or composition of offspring, the sex of the embryo was also detected by the genetic sex marker method. The results showed that the progeny of the control female was composed of male and female as expected, whereas the progenies of the neo-female were only males (**Figure 2C**). It was proven that the offspring of these neo-females was an all-male population. Therefore, that was to say, *MroDmrt11E* gene silencing in male PL period caused complete and functional sex reversal into neo-female, which could be applied to produce an all-male population.

Furthermore, with regard to the sex reversal induction and neo-female production in *M. rosenbergii*, both the manipulations of the single gene knockdown and AG ablation have crucial effects on male sexual differentiation. The detailed results of various gene silencing, such as *MroDmrt11E, Mr-IAG, and Mr-IR*, and AG ablation were summarized and compared in **Table 1**.

**Table 1:** The summary of sex reversal induction and neo-female production in *Macrobrachium rosenbergii*.

Manipulation	Animals	Methods and Schemes	Amount of Prawn	Survival prawn (Survival Ratio)	Gender of Prawn	Amount of Neo-Females (Sex Reversal ratio)	Reference
*MroDmrt11E* silencing	Individuals at postlarval stage (PL 15–30)	dsRNA (5 µg/individual) injection every month for 90 days	200	69 (34.50%)	33M + 22F + 14N	14 (29.79%)	The present study
*Mr-IAG* silencing	Males at postlarvae (PL 30)	dsRNA (5 µg/g body weight) injection twice a week for 71 injections over 9 months	19	10 (52.63%)	6M + 4N	4 (40%)	(3)
*Mr-IAG* silencing	Individuals at postlarval stage (PL 10)	dsRNA (4 µg/g body weight) injection every 5 days for 50 days	100	8 (8.00%)	3M + 2F + 3N	3 (50.00%)	(10)
*Mr-IAG* silencing	Individuals at postlarval stage (PL 10)	siRNA (0.5 µg/g body weight) injection every 5 days for 50 days	100	6 (6.00%)	3M + 1F + 2N	2 (40.00%)	(10)
*Mr-IR* silencing	Individuals at postlarval stage (PL 10)	dsRNA (4 µg/g body weight) injection every 5 days for 50 days	100	/	/	2 (not given)	(11)
*Mr-IR* silencing	Individuals at postlarval stage (PL 10)	siRNA (0.5 µg/g body weight) injection every 5 days for 50 days	100	/	/	1 (not given)	(11)
AG ablation	Males (PL 25–60)	Andrectomy, then 30-day cultivation	1,940	1,280 (65.98%)	878M + 402N	26 (1.33%)	(30)
AG ablation	All-male population (PL 20–30)	Andrectomy, then 30-day cultivation	4,137	2,718 (65.69%)	1989M + 729N	729 (17.62%)	(30)

M, males; F, females; N, neo-females.

### 3.3 The Comparative Transcriptomic Analysis of *MroDmrt11E* Knockdown

#### 3.3.1 The Differential Gene Expression Analysis of *MroDmrt11E* Silencing

To ascertain the effect of *MroDmrt11E* knockdown on the expression level of related genes or factors in sexual differentiation, comparative transcriptomic profiling from juvenile males of *M. rosenbergii* after *MroDmrt11E* silencing was enriched in this study. The transcriptomic libraries obtained about 50.74 million clean reads and 7.61G clean bases in *MroDmrt11E* RNAi group and 55.68 million clean reads and 8.35G clean bases in GFP RNAi group. The values of Q20 and Q30 were more than 97% and 93% in both groups, respectively.

Firstly, differential gene expression (DGE) analysis between *MroDmrt11E* silencing and control group generated 1,060 genes, of which 424 unigenes were upregulated and 636 were downregulated. Secondly, to assess how *MroDmrt11E* participates in the sex determination and/or sexual differentiation cascades in *M. rosenbergii*, the upregulated and downregulated unigenes with high percentages, which belong to the categories of response to various pathways, were identified. The top 10 representative groups of pathways with a higher percentage of differentially regulated genes were clustered into the phagosome, Phosphatidylinositide 3-kinases and protein kinase B (PKB also termed Akt) (PI3K-Akt) signaling pathway, viral carcinogenesis, cell cycle, pathogenic *Escherichia coli* infection, focal adhesion, apoptosis, RNA transport, estrogen signaling pathway, gap junction, and Rap1 signaling pathway. Meanwhile, the highest numbers of upregulated and downregulated unigenes were focused on zinc finger/zinc knuckle, and the higher amounts of differentially regulated genes were enriched on WD domain/G-beta repeat, tubulin/FtsZ gene family, RNA recognition motif, ribosomal protein, mitochondrial proteolipid, integrase core domain, and ATP synthase.

**Figure 3:**
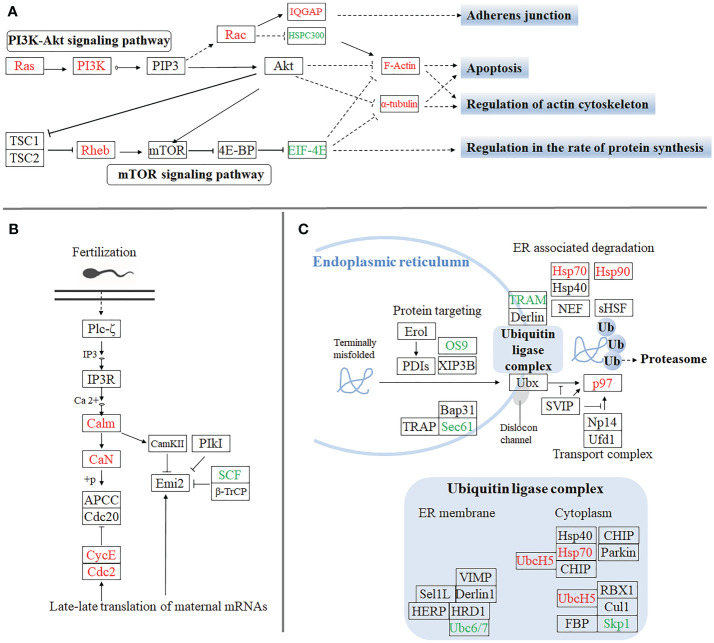
The significant differentially expressed unigenes enriched KEGG pathway analysis by the comparative transcriptomic analysis of *MroDmrt11E* knockdown. Four kinds of categories with a higher percentage of upregulated and downregulated transcripts were primarily focused on the pathways of apoptosis and regulation of actin cytoskeleton **(A)**, oocyte meiosis **(B)**, and protein processing in endoplasmic reticulum **(C)**, respectively. The primary upregulated transcripts (red color) and the downregulated transcripts (green color) were briefly profiled in the various signaling pathways.

Furthermore, to gain insight into the biological processes being operative during the sexual differentiation in *M. rosenbergii* including other differentially expressed genes, we have subjected transcripts (with significant differential expression) to DEG-enriched KEGG pathway analysis. A brief illustration of these significant differentially expressed genes, which come from the four categories of the enriched KEGG pathway analysis, was detailedly summarized (**Figure 3**). These four kinds of categories with a higher percentage of upregulated and downregulated unigenes were primarily focused on the pathways of apoptosis (**Figure 3A**), regulation of actin cytoskeleton (**Figure 3A**), oocyte meiosis (**Figure 3B**), and protein processing in the endoplasmic reticulum (**Figures 3A, C** **)**, respectively. Of these genes differentially upregulated and downregulated, the expression pattern of important genes playing crucial roles in these pathways was listed in **Table 1S**.

#### 3.3.2 The Response of Sex-Related Candidate Genes to *MroDmrt11E* Silencing

**Figure 4:**
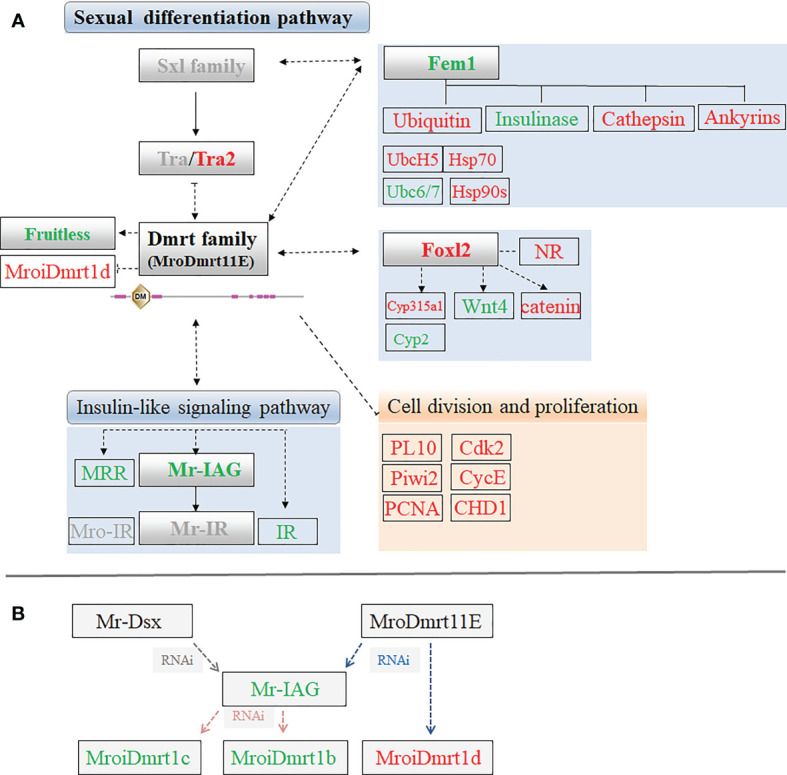
**(A)** The sex-related candidate unigenes enriched in putative sexual differentiation pathway by the comparative transcriptomic analysis of *MroDmrt11E* knockdown. The primary upregulated transcripts (red color) and the downregulated transcripts (green color) were briefly profiled in the signaling pathway. The genes with gray color possibly participated in the sexual differentiation but did not show significant differential expression by *MroDmrt11E* knockdown. **(B)** The brief relationship among Dmrt gene family and Mr-IAG was summarized to evaluate their potential influences involved in the sexual differentiation of *M. rosenbergii*.

To explore the effect of *MroDmrt11E* on the expression level of sex-related genes or factors, some significant differentially expressed unigenes were identified and enriched in the comparative transcriptomic profiling of *M. rosenbergii.* The influences or changes of these upregulated and downregulated transcripts were presented in **Table 2**. Furthermore, according to these differentially expressed transcripts involved in sexual differentiation or cell proliferation signal, the putative intuitive cascade regulation axis or networks of pathways were illustrated (**Figure 4A**).

**Table 2:** Significantly differential expression of sex-related candidate genes in sexual differentiation and gonad development.

Gene Description	NR ID	HIT Species	NRE-value	Identity	Gene ID	MR	GR	log2Foldchange	P value	Q value	Expression
Transformer-2b (*Tra2*)	QBY91827.1	*Macrobrachium rosenbergii*	8.00E-64	100.00%	Cluster-19843.19254	233.30	159.73	0.55	4.40E-03	8.30E-02	up
Zinc finger (fruitless homolog)	XP_037797540.1	*Penaeus vannamei*	1.00E-156	59.95%	Cluster-19843.21827	36.18	61.07	-0.76	2.97E-03	6.07E-02	down
Doublesex and mab-3 related transcription factor 1d (*MroiDmrt1d*)	MK468652.1	*Macrobrachium rosenbergii*	0.00E+00	100.00%	Cluster-19843.21058	94.54	25.51	1.89	1.43E-09	1.35E-07	up
Vitellogenin	BAD11098.1	*Pandalus hypsinotus*	0.00E+00	57.77%	Cluster-19843.27812	7.69	31.69	-2.04	2.60E-05	1.02E-03	down
Forkhead box L2 (*Foxl2*)	ALD48735.1	*Procambarus clarkii*	2.00E-109	62.80%	Cluster-19843.29432	64.02	24.86	1.36	1.29E-04	4.19E-03	up
Wnt family member 4 (*Wnt4*)	QBS32932.1	*Eriocheir sinensis*	1.00E-157	89.39%	Cluster-19843.33298	1.17	18.91	-4.02	1.05E-05	4.72E-04	down
Catenin alpha	XP_023712277.1	*Cryptotermes secundus*	0.00E+00	83.57%	Cluster-19843.15216	178.04	118.18	0.59	6.28E-03	1.10E-01	up
Nuclear receptor HR4 (*NR*)	XP_037795474.1	*Penaeus monodon*	0.00E+00	78.55%	Cluster-19843.1335	31.22	2.61	3.58	3.64E-07	2.30E-05	up
Cytochrome P450 (*CYP315a1*)	QBJ27553.1	*Sagmariasus verreauxi*	0.00E+00	61.01%	Cluster-19843.6938	36.59	10.26	1.83	2.31E-04	6.97E-03	up
Cytochrome P450 (*CYP2*)	ALA09303.1	*Eriocheir sinensis*	4.00E-173	48.21%	Cluster-19843.12355	0.71	5.17	-2.87	4.20E-02	4.58E-01	down
Feminization-1A (*Fem1*)	AKS25864.1	*Eriocheir sinensis*	0.00E+00	81.29%	Cluster-19843.18428	51.97	90.19	-0.80	1.78E-04	5.56E-03	down
Insulin-degrading enzyme-like (*Insulinase*)	XP_027229987.1	*Penaeus vannamei*	0.00E+00	75.45%	Cluster-19843.21622	28.19	61.21	-1.12	8.09E-05	2.79E-03	down
Polyubiquitin-C	XP_023158674.1	*Ceratitis capitata*	0.00E+00	99.77%	Cluster-19843.22200	520.82	295.05	0.82	1.91E-11	2.35E-09	up
Ubiquitin-conjugating enzyme E2 (*UbcH5*)	XP_027238029.1	*Penaeus vannamei*	3.00E-93	96.60%	Cluster-19843.21815	489.46	234.66	1.06	3.91E-17	8.67E-15	up
Ubiquitin-conjugating enzyme E2 (*Ubc6/7*)	XP_037787246.1	*Penaeus monodon*	2.00E-121	80.93%	Cluster-19843.21559	45.40	99.23	-1.13	4.44E-07	2.72E-05	down
Insulin-like androgenic gland specific factor (*Mr-IAG*)	FJ409645.1	*Macrobrachium rosenbergii*	7.00E-171	100.00%	Cluster-19843.27103	41.70	192.24	-2.20	2.55E-27	1.11E-24	down
Insulin receptor (*IR*)	CDI30232.1	*Blattella germanica*	3.00E-171	32.11%	Cluster-19843.16620	5.26	20.60	-1.97	9.04E-04	2.23E-02	down
Male reproductive-related protein (*MRR*)	ABQ41253.1	*Macrobrachium rosenbergii*	2.00E-30	96.38%	Cluster-19843.13975	17.51	92.49	-2.40	3.56E-15	6.73E-13	down
											
Piwi-like protein Ago3 (*Piwi2*)	AZN25269.1	*Penaeus monodon*	0.00E+00	65.67%	Cluster-19843.21923	634.88	314.25	1.01	4.12E-20	1.16E-17	up
Putative germ-line specific RNA helicase vasa protein (PL10-like protein, *PL10*)	ADB28896.1	*Macrobrachium nipponense*	0.00E+00	91.28%	Cluster-19843.21825	618.27	349.97	0.82	2.39E-13	3.61E-11	up
Proliferating cell nuclear antigen (*PCNA*)	AYT70175.1	*Rimicaris exoculata*	8.00E-122	92.63%	Cluster-19843.18613	236.89	115.48	1.04	1.02E-08	8.23E-07	up
Chromodomain-helicase-DNA-binding protein 1-like (*CHD1*)	XP_037772797.1	*Penaeus monodon*	0.00E+00	85.94%	Cluster-19843.19508	161.64	114.79	0.49	3.66E-02	4.18E-01	up
Cyclin-dependent kinases 2 (*Cdk2*)	AVM39148.1	*Macrobrachium nipponense*	0.00E+00	99.67%	Cluster-19843.21750	880.36	387.44	1.18	6.33E-36	4.03E-33	up
*Cyclin E*	AGW23550.1	*Penaeus monodon*	0.00E+00	78.86%	Cluster-19843.23974	222.12	90.75	1.29	1.09E-11	1.38E-09	up
*cathepsin B*	AEC22812.1	*Macrobrachium nipponense*	0.00E+00	95.53%	Cluster-19843.21212	878.83	377.00	1.22	7.89E-38	5.44E-35	up
*cathepsin L*	AGN52717.1	*Macrobrachium rosenbergii*	0.00E+00	99.61%	Cluster-19843.22524	4110.23	1594.17	1.37	1.19E-207	9.99E-204	up
*cathepsin-D*	AMQ98967.1	*Macrobrachium rosenbergii*	0.00E+00	99.46%	Cluster-19843.27411	418.37	163.43	1.36	2.13E-22	7.24E-20	up

MR, MroDmrt11E RNAi Readcount; GR, GFP RNAi Readcount.

In addition, the proven switch gene of sexual differentiation, *Mr-IAG*, was found in a downregulated transcript, but another member of *Dmrt* gene family, *MroiDmrt1d*, was identified as an upregulated transcript in the transcriptomic of *MroDmrt11E* knockdown. The possible relationships among *Mr-Dsx*, *MroDmrt11E*, *Mr-IAG*, and other *Dmrt* gene families were summarized based on the known information of the various RNAi results of these genes. Dmrt gene family probably participated in the transcriptional activation of Mr-IAG and possibly played great roles in the switch of Mr-IAG-related regulatory signaling of male differentiation. Moreover, Mr-IAG directly or indirectly influenced the expression of partial genes of Dmrt family (**Figure 4B**).

## 4 Discussion

### 4.1 The Regulation Relationship Between *Dmrt* Gene Family and Insulin Signaling Pathway in Sexual Differentiation

*MroDmrt11E* is a testis-prominent expressed gene and is speculated to play an important role in the developmental processes of sexual differentiation (26). The present study set out with the aim of supplying direct evidence from animal experiments and exploring the biological function of *MroDmrt11E* involving sexual differentiation. In addition, Dmrts were shown relative to IAG and involved in sexual differentiation (25, 28). But the molecular mechanism of Dmrts regulating sexual differentiation is little known. The position of the Dmrt gene family in the signaling pathways orchestrating sexual differentiation was also expounded by comparative transcriptomic analysis of *MroDmrt11E* knockdown in *M. rosenbergii*.

Firstly, the important highlight of this study has given remarkable evidence that *MroDmrt11E* played a crucial role in male sexual differentiation in *M. rosenbergii* and provided a novel sexual regulation illustration of the conserved *Dmrt* gene family in crustaceans. Although the crucial role of IAG in orchestrating male sexual differentiation has been characterized and the production of all monosex progenies on IAG or IAG’s receptor manipulation has been achieved, limited information on conserved genes involved in sexual differentiation has been published to date (13). In the present study, a gene silencing strategy was implemented to expound whether *MroDmrt11E* participated in male sexual differentiation of *M. rosenbergii*. Significantly, *MroDmrt11E* silencing in juvenile males successfully caused the disappearance of male characteristics (such as male genital pores and male reproductive system) while promoting the formation and development of female features and inducing full functional sex reversal individuals. Moreover, the neo-female individuals gave rise to ovarian maturity, induced appearance features of females, and were utilized to produce all-male populations.

Secondly, apart from the characterization of Dmrt itself, little information is available regarding key factors upstream or downstream of the Dmrt gene family and other elements of the signal transduction pathway involving sexual differentiation (27). Thus, we focused on the relationship between the Dmrt gene family and Mr-IAG. On one hand, it is reported that *in vivo* silencing of the *Mr-IAG* gene significantly decreased the expression of two *MroDmrt* genes, *MroiDmrt1b* and *MroiDmrt1c*, thereby suggesting the possible role of these two genes in the IAG switch and sexual differentiation processes (27). On the other hand, gene knockdown of *Mr-Dsx* resulted in a pronounced suppression of the *insulin-like androgenic hormone (Mr-IAG)* gene, which indicated that Mr-Dsx may participate in the transcriptional activation of *IAG* (28). In the present study, knockdown of *MroDmrt11E* was proven continuously related to the decrease of the *Mr-IAG* transcript. However, *MroiDmrt1d* was contrarily found upregulated in the male of *MroDmrt11E* knockdown, which meant *MroDmrt11E* caused pronounced suppression of MroiDmrt1d. Thus, a complex regulation network among the Dmrt gene family and Mr-IAG was summarized. In brief, both Mr-Dsx and MroDmrt11E participate in the transcriptional activation of Mr-IAG, but Mr-IAG is involved in the activation of MroiDmrt1b and MroiDmrt1c. These results implied that a feedback loop of the Dmrt gene family may exist. And, the various members of the Dmrt gene family had different effects on the expression of *Mr-IAG* gene by directly or indirectly influencing the switch of male sexual differentiation in *M. rosenbergii* (25, 28).

Differently, *MniDMRT11E* knockdown significantly increased *insulin-like androgenic gland factor* expression in the oriental river prawn *M. nipponense* (25), which was contrary to the result of the *MroDmrt11E* knockdown sustainedly repressing the expression of *Mr-IAG*. It indicated that the evolutionarily conserved *Dmrt* gene family plays widely divergent roles in the sexual differentiation of crustaceans (25, 28).

Moreover, an *insulin-like receptor homolog and a male reproductive-related protein (MRR)* were found significantly downregulated from the transcriptomic analysis of *MroDmrt11E* knockdown. *Insulin-like receptor (IR)* functions as the pivotal member of the insulin family signaling pathway and directs the sexual differentiation in mammals (31) and some aquatic livestock (32, 33). The binding of the insulin-like peptide ligand initiates a cascade of phosphorylation events, stimulating the downstream signal transduction and resulting cellular effect (31). Earlier evidence has shown that IR interacts with IAG to regulate sexual differentiation and spermatogenesis in crustacean (5, 11, 33, 34). In more detail, the insulin-like receptor, termed Mr-IR (33), functioned as a receptor for Mr-IAG, and its knockdown induced sex reversal and retarded the process of spermatogenesis in *M. rosenbergii* (11). In this study, not *Mr-IR* but a novel *insulin-like receptor homolog* was found as a significantly downregulated transcript, which indicated that more than one IR are involved in the insulin family signaling pathway and participate in the regulation of sexual differentiation. Meanwhile, a male reproductive-related protein expressed in the terminal ampullae of the male reproductive system (35) was also identified as a significantly downregulated transcript. It means that *MroDmrt11E* was required for the transcriptional activation of Mr-IAG or IR or MRR, but it was not clear whether the *MroDmrt11E* gene can directly stimulate their expressions in the prawn.

In brief, this study revealed the potential and complex regulatory relationships among various *Dmrt *gene families and several insulin signaling pathway-related factors. It also implied that the insulin signal pathway is crucial for female/male reproductive development or maintenance in crustaceans.

### 4.2 Sex-Related Candidate Genes Involved in the *MroDmrt11E* Knockdown

*MroDmrt11E* has been validated to affect the development of male gonads and played its potential function in sexual differentiation and reproductive development (26). Compared with differently expressed genes of the transcriptomic library, a great deal of sex-related candidate genes were focused on. However, due to the limitation of the molecular mechanism of sexual differentiation in crustaceans, the position of the *MroDmrt11E* or Dmrt gene family in the signaling pathways orchestrating sexual differentiation of *M. rosenbergii* is hard to be clarified (28). Recently, with the analogous transcripts addressed in many crustaceans, it suggested that crustacean species may adopt a similar sex determination and/or sexual differentiation pathway to that reported in insects (36). Therefore, the well-characterized molecular mechanism of sex determination of insects is used as an important reference clue. For instance, the primary sex determination and somatic sexual differentiation are controlled by a genetic hierarchy X:A > Sex-lethal (Sxl) > Transformer/Transformer 2 (Tra/Tra2) > Dsx and Fruitless (Fru) in *Drosophila* (16, 21, 37, 38). Therefore, in the present study, some sex-related homologs, including *Tra2, Foxl2, Fru, Feminization-1 (Fem-1)*, were identified and screened as significant differential genes from the comparative transcriptomic library. In the following sections, we tried to provide more valuable information and infer the potential roles of these candidate sex-related genes in sex determination and/or sexual differentiation pathways.

#### 4.2.1 Transformer and Transformer-2

Tra and Tra-2 are mRNA splicing factors and act as a downstream splicing complex, regulating specific splicing of target RNAs (21, 37–39). In *Drosophila* species, it is known that the action of Sxl results in the active splice variant of Tra in females (37). Then, Tra2 acts in concert with Tra to regulate female-specific splicing of target RNAs (21, 37, 38). In the present study, upregulated *Tra*2 homolog was identified in the transcriptomic library of *MroDmrt11E* silencing, indicating that *MroDmrt11E* possibly participated in the regulation of sex or gonad-specific splicing of target RNAs through suppression of the expression of *Tra2*. On the other hand, four Sxl isoforms, named MroSxl1, MroSxl2, MroSxl3, and MroSxl4, have been identified in both males and females in *M. rosenbergii* (40). However, none of these *Sxl* genes were screened as significantly differential transcripts from the comparative transcriptomic library until now. In addition, several *Sxl* and *Tra-2* isoform-encoding transcripts show broad tissue expression but not sex-biased expression patterns in crustaceans, suggesting that Sxl-Tra-Dsx may be unique to Drosophilidae and not conserved among decapods (41).

#### 4.2.2 Fruitless

Fru, defined by its zinc fingers and forkhead box L2 (Foxl2) defined by a unique DNA-binding domain, is known as a transcription factor (20, 21, 37, 38). *Fru* is a male-promoting gene, regulating the development of the male central nervous system and male sexual behavior (39). It is spliced in the absence of the female Tra/Tra-2 complex, carried out by non-sex-specific splicing machinery (21, 37, 38). Nevertheless, *Fru* showed sexual dimorphism in ovaries and testis, which displayed upregulated expression in ovaries of *M. rosenbergii* in the comparative transcriptomic database of gonads (42).

On the other hand, *Foxl2* expression ensues and acts to inhibit the male pathway while promoting the female pathway through the action of Rspo1, Wnt family member 4 (Wnt4), and catenin in the absence of the Sry-driven expression of *Sox9* (43). Interestingly, Foxl2 displayed upregulated expression in testis from the transcriptomic database of gonads of *M. rosenbergii* (42). Meanwhile, *Sp-Wnt4*, a member of *Wnt4* gene in mud crab, *Scylla Paramamosain*, was expressed at a higher level in the ovary compared to other tissues, but the expression level of *Sp-Wnt4* was significantly increased in testis after unilateral eyestalk ablation (44). In the present study, significantly upregulated *Foxl2*, downregulated *Fru*, and *Wnt4* homolog were identified in *MroDmrt11E* knockdown. It suggested that *MroDmrt11E* expression might act to inhibit the expression of *Foxl2* gene while promoting the action of Fru and Wnt4 in the sexual differentiation process.

In addition, it is reported that Foxl2 was able alone or with nuclear receptor subfamily 5 group A member 2 (Nr5a2) jointly to upregulate the expression of *cyp19a* and repress the expression of *Dmrt1* in the olive flounder *Paralichthys olivaceus* (45). It meant that Foxl2 may play an important role in ovarian differentiation by maintaining *cyp19a* expression and antagonizing the expression of *Dmrt1* (45). The cytochrome *P450 CYP315a1*, a member of the CYP superfamily gene, was implicated in the ecdysteroidogenic pathway (46). Meanwhile, *CYP315a1* was also identified as a sex-related gene. From the comparative transcriptome analysis of *M. rosenbergii*, CYP315a1 was suggested involved in testicular development (42). Nuclear receptors are a class of proteins found within cells that are responsible for sensing steroid and thyroid hormones and certain other molecules (47, 48). Steroid hormones are well known to be responsible for controlling reproduction and development in higher organisms like arthropods (46, 47). In the present study, upregulated *Foxl2, nuclear receptor (NR)*, and *CYP315a1* homologs in *MroDmrt11E* knockdown were all identified, and the expression of their transcripts was by the above studies, which indicated that *MroDmrt11E* inhibited the expression of *CYP315a1* through repressing the expression of *Foxl2* and/or *NR*. Moreover, *MroDmrt11E* perhaps participated in the signaling pathway of various hormones and played great roles in reproduction and gonad differentiation.

#### 4.2.3 Feminization-1

Fem-1 is characterized by one of the most common protein–protein interaction motifs and ankyrin repeat motifs and known as a regulatory factor of signal transduction in the sex determination signaling pathway (21, 37, 38, 49). In *M. rosenbergii*, *Mrfem-1* was exclusively expressed in the ovary, suggesting that Mrfem-1 might be associated with ovarian maturation in prawn (50). An ovary-specific gene Fem-1 homolog, *Mnfem-1*, has been identified in the oriental river prawn, *M. nipponense* (51). The Mnfem-1 protein can be potentially interactive with cathepsin L and proteins containing the domains of insulinase (also called an insulin-degrading enzyme), ankyrin, or ubiquitin in yeast two hybridization (51). Accordantly, in the present study, downregulated *Fem-1A* and *insulinase* homolog, three upregulated cathepsin homologs, and one *polyubiquitin-C* homolog were identified in the comparative transcriptomic analysis of *MroDmrt11E* knockdown (**Table 2**). Meanwhile, several significant differentially expressed genes encoding the domains of ankyrin were found as upregulated transcripts (data not shown). It suggested that *MroDmrt11E* probably played significant roles in activating or inhibiting the expression of these genes or elements (such as cathepsin, insulinase, and ubiquitin) through suppression of the Fem-1 homolog.

Cathepsins, ubiquitously present in most organisms, belong to a family of proteases that cleaves other proteins, and some of these genes were shown to be involved in the ovarian maturation and embryo development of *Penaeus japonicus* (52) and *M. nipponense* (53). Furthermore, the gonadal transcriptomic database of *Oratosquilla oratoria* included eight cathepsin genes (54). Two *cathepsins, cathepsin I and cathepsin D-like*, were predominantly expressed in the testis of *O. oratoria*, suggesting that cathepsins may be involved in testicular development (54). In the present study, three upregulated cathepsin homologs were identified in the comparative transcriptomic analysis of *MroDmrt11E* knockdown, which indicated that *MroDmrt11E* may play an important role in sexual and/or gonad differentiation by antagonizing the expression of *cathepsins*.

#### 4.2.4 Heat Shock Protein

Heat shock protein (Hsp) contributes to the interaction with steroid hormone receptors, temperature, estrogen signaling, etc., and several Hsps are critical for successful embryogenesis and reproduction (55, 56). It is reported that three *Hsps (heat shock protein 27, heat shock protein 70, and heat shock protein 70 cognate3)* exhibited upregulated expression patterns in the transcriptomic database of testis from *M. rosenbergii* (42). In the present study, four *heat shock protein 90 homologs (Hsp90s) *and one *Hsp70* homolog were identified as upregulated transcripts, respectively. It was indicated that *MroDmrt11E* probably had a suppression effect on the expressions of *Hsp90s* and *Hsp70*, which may play a regulatory role in spermatogenesis of testis and gonad development.

In conclusion, many highly conserved sex-related candidate homologs were identified as upregulated or downregulated transcripts in the comparative transcriptomic library of *M. rosenbergii* testis after *MroDmrt11E* knockdown. Although these data were screened from the transcriptomic library and need to be further examined, this study provided new information on the sex-related candidate genes and their possible regulation mechanism in sexual differentiation. Notwithstanding its limitation, the putative position of *MroDmrt11E* involved in the signaling pathways orchestrating sexual differentiation of *M. rosenbergii* is worth further clarifying.

### 4.3 *MroDmrt11E* Participated in Gonad Development in *M. rosenbergii*

The *MroDmrt11E* presented testis-prominent distribution and were localized in spermatogonia and spermatozoa during spermatogenesis, suggesting that the *MroDmrt11E* participated in the development of the male reproductive system (26). In the present study, several cell division and proliferation-related candidate genes, including *Piwi2, PL-10, PCNA, Cdk2, CycE*, and *CHD1* homologs, were found as upregulated genes that are previously implicated in regulating germ cells.

1. *De novo* transcriptomic analysis of the Japanese mantis shrimp *O. oratoria* showed that *Piwi2 and vasa-like protein* genes exhibited a higher expression level in ovaries than that in testis, suggesting that they may play an important role in germ cell differentiation (54).
2. Germline-specific vasa protein, also known as PL10-like protein, encodes an ATP-dependent RNA helicase belonging to the DEAD and is essential not only for germ cell specification during embryogenesis but also for the completion of meiosis of the germ cells in adults (57). A vasa-like gene, *Mrvlg*, is specifically expressed in the germ cells and differentially expressed during germ cell differentiation in both ovary and testis of *M. rosenbergii* (57).
3. Proliferating cell nuclear antigen (PCNA) is an evolutionarily well-conserved protein found in all eukaryotic species and plays an important role in cell cycle regulation and checkpoint control, DNA repair, translesion DNA synthesis, DNA methylation, chromatin remodeling, and gonadenogenesis in various species (58). The *PCNA* of Chinese shrimp *F. chinensis*, termed *FcPCNA*, was highly expressed in proliferating tissues, such as hematopoietic and ovary (59). In *O. oratoria*, *PCNA* was expressed at a higher level in the ovaries than in the testis, suggesting it may be related to oogenesis (36).

In conclusion, the upregulation of all of these cell division, proliferation, and differentiation-related candidate homologs suggested that *MroDmrt11E* probably participates in regulating both spermatogenesis and oogenesis. Despite this preliminary characteristic, this study indicated the important clues for further functional research of *MroDmrt11E* in gonad development.

### 4.4 The Changes of Primary Kyoto Encyclopedia of Genes and Genomes Pathways in *MroDmrt11E* Silencing

*MroDmrt11E* silencing induced dramatic sex-related alterations, including male feature feminization, extensive male reproductive system inhibition, oocyte meiosis promotion, and the ovarian development of neo-females. The phenomenon of complete and functional sex reversal individuals, induced by *MroDmrt11E* knockdown, showed a close relationship with various biological pathways. The pathway enrichment was used to capture the functional specialization of *MroDmrt11E* silencing and, based on known functional roles, validated various biological aspects in prawn and other closely related crustaceans.

Firstly, the pathway of protein processing, which happens in the endoplasmic reticulum (ER), was thought to be significantly involved in energy and substance metabolism during sexual differentiation and sex reversal. Abundant transcripts were exposed to protein processing in ER, including upregulated genes, such as *Sec62/63, Sec61, Hsp70, Hsp90, P97*, and *UbcH5,* and downregulated genes, such as *Ubc6/7, GlcII, OS9, TRAM*, and *Skp1*. Meanwhile, the mammalian target of rapamycin (mTOR) signaling pathway is also involved in the regulation of the rate of protein synthesis. The upregulated *Rheb* gene and downregulated *EIF-4E* gene were enriched. In addition, a series of upregulated factors such as *PI3K, Rac, F-Actin*, and *α-tubulin*, involved in PI3K/Akt signaling, were intensively activated and participated in the regulation of actin cytoskeleton and apoptosis. It was reported that the transcription of *MroDmrt11E* was prominent in testis and localized in spermatogonia and spermatozoa during spermatogenesis (26). The degeneration of the male reproductive system, induced by *MroDmrt11E* silencing in PL males, indicated that *MroDmrt11E* was a crucial regulator of testicular formation. The absence of male characteristics (male genital pores and reproductive system) in juvenile males probably had close relationships with the pathway of apoptosis and regulation of actin cytoskeleton, which is possibly involved in the development and reconstruction of the female reproductive system and ovary.

Moreover, the enrichment of KEGG’s oocyte meiosis pathway indicates its involvement in the ovarian development of neo-females. Several factors, upregulated genes, namely, *Calm, CaN, CycE*, and *Cdk2*, and one downregulated gene, *SFC*, were involved in the oocyte meiosis. Most genes downstream of the fertilization and translation of maternal mRNAs were upregulated, indicating *MroDmrt11E* plays an important role in the regulation of oocyte meiosis. Furthermore, many upregulated factors, such as *Piwi2* (36), *PL-10* (57), *PCNA* (58), *Cdk2* (60), *CycE*, and *CHD1* homologs, were all intensively activated to promote the germ cell proliferation and cell differentiation of the ovarian development by *MroDmrt11E* knockdown. These significant responses to primary KEGG pathways provided clues to understand the mechanism of sexual differentiation.

In conclusion, dsRNA-mediated gene knockdown of *MroDmrt11E* led to a complete and functional sexual reversal, which possibly established a crucial basis for the new development of manipulating monosex progeny of prawn. The data of comparative transcriptomic analysis, after *MroDmrt11E* silencing mediated, provided a deeper understanding of the molecular regulatory network underlying sexual differentiation in *M. rosenbergii* and provided a novel insight into the roles of Dmrt homologs in the sexual differentiation of crustaceans.

## Data Availability Statement

The original contributions presented in the study are included in the article/Supplementary Material. RNA-sequencing data are available through the NCBI Sequence Read Archive (SRA) Database (https://www.ncbi.nlm.nih.gov/Traces/sra/), under BioProject Number: PRJNA796284. Further inquiries can be directed to the corresponding author.

## Ethics Statement

The authors followed all applicable international, national, and/or institutional guidelines for the care and use of animals.

## Author Contributions

W-MM conceived and designed the study and analyzed most of the data. H-JX, Y-LC, Y-MW, J-YL, J-WL, and S-QS performed the experiments. J-SY developed the idea for the study. All authors were involved in writing and approving the article.

## Funding

This work was supported by the National Key Research and Development Program of China (grant number 2018YFD0900200), the National Natural Science Foundation of China (grant number 31872545), and the Top Disciplines of Zhejiang Province, Biology engineering (grant numbers ZS2021005 and ZS2021021).

## Conflict of Interest

The authors declare that the research was conducted in the absence of any commercial or financial relationships that could be construed as a potential conflict of interest.

## Publisher’s Note

All claims expressed in this article are solely those of the authors and do not necessarily represent those of their affiliated organizations, or those of the publisher, the editors and the reviewers. Any product that may be evaluated in this article, or claim that may be made by its manufacturer, is not guaranteed or endorsed by the publisher.

## References

[1] Shen SQ Li JW Xu HJ Yang JS Ma WM Qian GY. Sexual Characteristic Development and Sex Identification of Juvenile Prawns, *Macrobrachium Rosenbergii* . Aquac Res (2020) 51:3718–28. doi: 10.1111/are.14721

[2] Ventura T Manor R Aflalo ED Weil S Rosen O Sagi A. Timing Sexual Differentiation: Full Functional Sex Reversal Achieved Through Silencing of a Single Insulin-Like Gene in the Prawn, Macrobrachium Rosenbergii. Biol Reprod (2012) 86:1–13. doi: 10.1095/biolreprod.111.097261 22133694

[3] Ventura T Manor R Aflalo ED Weil S Khalaila I Rosen O. Expression of an Androgenic Gland-Specific Insulin-Like Peptide During the Course of Prawn Sexual and Morphotypic Differentiation. ISRN Endocrinol (2011) 2011:1–11. doi: 10.5402/2011/476283 PMC3262648 22363879

[4] Aflalo ED Dandu RVSN Verghese JT Rao N Samraj TYC Ovadia O. Neo-Females Production and All-Male Progeny of a Cross Between Two Indian Strains of Prawn (*Macrobrachium Rosenbergii*): Population Structure and Growth Performance Under Different Harvest Strategies. Aquaculture (2014) 428–9:7–15. doi: 10.1016/j.aquaculture.2014.02.021

[5] Manor R Weil S Oren S Glazer L Aflalo ED Ventura T. Insulin and Gender: An Insulin-Like Gene Expressed Exclusively in the Androgenic Gland of the Male Crayfish. Gen Comp Endocrinol (2007) 150:326–36. doi: 10.1016/j.ygcen.2006.09.006 17094989

[6] Rosen O Manor R Weil S Gafni O Linial A Aflalo ED. A Sexual Shift Induced by Silencing of a Single Insulin-Like Gene in Crayfish: Ovarian Upregulation and Testicular Degeneration. PloS One (2010) 5:1–10. doi: 10.1371/journal.pone.0015281 PMC3000327 21151555

[7] Mareddy VR Rosen O Thaggard HB Manor R Kuballa AV Aflalo ED. Isolation and Characterization of the Complete cDNA Sequence Encoding a Putative Insulin-Like Peptide From the Androgenic Gland of Penaeus Monodon. Aquaculture (2011) 318:364–70. doi: 10.1016/j.aquaculture.2011.05.027

[8] Ventura T Rosen O Sagi A. From the Discovery of the Crustacean Androgenic Gland to the Insulin-Like Hormone in Six Decades. Gen Comp Endocrinol (2011) 173:381–8. doi: 10.1016/j.ygcen.2011.05.018 21679714

[9] Ventura T Manor R Aflalo ED Weil S Raviv S Glazer L. Temporal Silencing of an Androgenic Gland-Specific Insulin-Like Gene Affecting Phenotypical Gender Differences and Spermatogenesis. Endocrinology (2009) 150:1278–86. doi: 10.1210/en.2008-0906 18988670

[10] Tan K Zhou M Jiang H Jiang D Li Y Wang W. siRNA-Mediated MrIAG Silencing Induces Sex Reversal in *Macrobrachium Rosenbergii* . Mar Biotechnol (2020) 22. doi: 10.1007/s10126-020-09965-4 32337657

[11] Tan K Li Y Zhou M Wang W. siRNA Knockdown of MrIR Induces Sex Reversal in Macrobrachium Rosenbergii. Aquaculture (2020) 523:735172. doi: 10.1016/j.scitotenv.2020.140326 32337657

[12] Levy T Sagi A. The “IAG-Switch”—A Key Controlling Element in Decapod Crustacean Sex Differentiation. Front Endocrinol (Lausanne) (2020) 11:651. doi: 10.3389/fendo.2020.00651 33013714 PMC7511715

[13] Farhadi A Cui W Zheng H Li S Zhang Y Ikhwanuddin M. The Regulatory Mechanism of Sexual Development in Decapod Crustaceans. Front Mar Sci (2021) 8:679687. doi: 10.3389/fmars.2021.679687

[14] Kopp A. Dmrt Genes in the Development and Evolution of Sexual Dimorphism. Trends Genet (2012) 28:175–84. doi: 10.1016/j.tig.2012.02.002.Dmrt PMC3350790 22425532

[15] Picard MA-L Cosseau C Mouahid G Duval D Grunau C Toulza È.. The Roles of Dmrt (Double Sex/Male-Abnormal-3 Related Transcription Factor) Genes in Sex Determination and Differentiation Mechanisms: Ubiquity and Diversity Across the Animal Kingdom. C R Biol (2015) 338:451–62. doi: 10.1016/j.crvi.2015.04.010 26043799

[16] Fujii T Shimada T. Sex Determination in the Silkworm, Bombyx Mori: A Female Determinant on the W Chromosome and the Sex-Determining Gene Cascade. Semin Cell Dev Biol (2007) 18:379–88. doi: 10.1016/j.semcdb.2007.02.008 17446095

[17] Toyota K Kato Y Sato M Sugiura N Miyagawa S Miyakawa H. Molecular Cloning of Doublesex Genes of Four Cladocera (Water Flea) Species. BMC Genomics (2013) 14:239. doi: 10.1186/1471-2164-14-239 23575357 PMC3637828

[18] Verhulst EC Van de zande L. Double Nexus-Doublesex Is the Connecting Element in Sex Determination. Brief Funct Genomics (2015) 14:396–406. doi: 10.1093/bfgp/elv005 25797692 PMC4652034

[19] Yi W Ross JM Zarkower D. Mab-3 Is a Direct Tra-1 Target Gene Regulating Diverse Aspects of C. Elegans Male Sexual Development and Behavior. Development (2000) 127:4469–80. doi: 10.1242/dev.127.20.4469 11003845

[20] Huang S Ye L Chen H. Sex Determination and Maintenance: The Role of DMRT1 and FOXL2. Asian J Androl (2017) 19:619–24. doi: 10.4103/1008-682X.194420 PMC5676419 28091399

[21] Millington JW Rideout EJ. Sex Differences in *Drosophila* Development and Physiology. Curr Opin Physiol (2018) 6:46–56. doi: 10.1016/j.cophys.2018.04.002

[22] Zhang EF Qiu GF. A Novel Dmrt Gene Is Specifically Expressed in the Testis of Chinese Mitten Crab, *Eriocheir Sinensis* . Dev Genes Evol (2010) 220:151–9. doi: 10.1007/s00427-010-0336-2 20809137

[23] Chandler JC Fitzgibbon QP Smith G Elizur A Ventura T. ). Y-Linked Idmrt1 Paralogue (iDMY) in the Eastern Spiny Lobster, Sagmariasus Verreauxi: The First Invertebrate Sex-Linked Dmrt. Dev Biol (2017) 430:337–45. doi: 10.1016/j.ydbio.2017.08.031 28864068

[24] Li S Li F Yu K Xiang J. Identification and Characterization of a Doublesex Gene Which Regulates the Expression of Insulin-Like Androgenic Gland Hormone in. Fenneropenaeus chinensis Gene (2018) 649:1–7. doi: 10.1016/j.gene.2018.01.043 29339074

[25] Wang Y Jin S Fu H Qiao H Sun S Zhang W. Identification and Characterization of the DMRT11E Gene in the Oriental River Prawn Macrobrachium Nipponense. Int J Mol Sci (2019) 20:1–17. doi: 10.3390/ijms20071734 PMC6480115 30965605

[26] Yu YQ Ma WM Zeng QG Qian YQ Yang JS Yang WJ. Molecular Cloning and Sexually Dimorphic Expression of Two Dmrt Genes in the Giant Freshwater Prawn, *Macrobrachium Rosenbergii* . Agric Res (2014) 3:181–91. doi: 10.1007/s40003-014-0106-x

[27] Amterat Abu Abayed F Manor R Aflalo ED Sagi A. Screening for Dmrt Genes From Embryo to Mature Macrobrachium Rosenbergii Prawns. Gen Comp Endocrinol (2019) 282:113205. doi: 10.1016/j.ygcen.2019.06.009 31201800

[28] Zhong P Zhou T Zhang Y Chen Y Yi J Lin W. Potential Involvement of a DMRT Family Member (Mr-Dsx) in the Regulation of Sexual Differentiation and Moulting in the Giant River Prawn Macrobrachium Rosenbergii. Aquac Res (2019) 00:1–13. doi: 10.1111/are.14262

[29] Jiang XH Qiu GF. Female-Only Sex-Linked Amplified Fragment Length Polymorphism Markers Support ZW/ZZ Sex Determination in the Giant Freshwater Prawn *Macrobrachium Rosenbergii* . Anim Genet (2013) 44:782–5. doi: 10.1111/age.12067 23763724

[30] Aflalo ED Hoang TTT Nguyen VH Lam Q Nguyen DM Trinh Q. A Novel Two-Step Procedure for Mass Production of All-Male Populations of the Giant Freshwater Prawn Macrobrachium rosenbergii. Aquaculture (2006) 256:468–78. doi: 10.1016/j.aquaculture.2006.01.03

[31] Hubbard SR Till JH. Protein Tyrosine Kinase Structure and Function. Annu Rev Biochem (2000) 69:373–93. doi: 10.1146/annurev.biochem.69.1.373 10966463

[32] Gricourt L Mathieu M Kellner K. An Insulin-Like System Involved in the Control of Pacific Oyster Crassostrea Gigas Reproduction: hrIGF-1 Effect on Germinal Cell Proliferation and Maturation Associated With Expression of an Homologous Insulin Receptor-Related Receptor. Aquaculture (2006) 251:85–98. doi: 10.1016/j.aquaculture.2005.05.015

[33] Sharabi O Manor R Weil S Aflalo ED Lezer Y Levy T. Identification and Characterization of an Insulin-Like Receptor Involved in Crustacean Reproduction. Endocrinology (2016) 157:928–41. doi: 10.1210/en.2015-1391 26677879

[34] Guo Q Li S Lv X Xiang J Sagi A Manor R. A Putative Insulin-Like Androgenic Gland Hormone Receptor Gene Specifically Expressed in Male Chinese Shrimp. Endocrinology (2018) 159:2173–85. doi: 10.1210/en.2017-03253 29596627

[35] Dai ZM Zhu XJ Yang WJ. Full-Length Normalization Subtractive Hybridization: A Novel Method for Generating Differentially Expressed cDNAs. Mol Biotechnol (2009) 43:257–63. doi: 10.1007/s12033-009-9198-0 19669953

[36] Yan H Cui X Shen X Wang L Jiang L Liu H. *De Novo* Transcriptome Analysis and Differentially Expressed Genes in the Ovary and Testis of the Japanese Mantis Shrimp Oratosquilla Oratoria by RNA-Seq. Comp Biochem Physiol - Part D Genomics Proteomics (2018) 26:69–78. doi: 10.1016/j.cbd.2018.04.001 29702368

[37] Pomiankowski A Nöthiger R Wilkins A. The Evolution of the Drosophila Sex-Determination Pathway. Genetics (2004) 166:1761–73. doi: 10.1534/genetics.166.4.1761 PMC1470811 15126396

[38] Sánchez L. Sex-Determining Mechanisms in Insects. Int J Dev Biol (2008) 52:837–56. doi: 10.1387/ijdb.072396ls 18956315

[39] Sun X Yang H Sturgill D Oliver B Rabinow L Samson ML. Sxl-Dependent, Tra/Tra2-Independent Alternative Splicing of the *Drosophila Melanogaster* X-Linked Gene Found in Neurons. G3 Genes Genomes Genet (2015) 5:2865–74. doi: 10.1534/g3.115.023721 PMC4683657 26511498

[40] Yu Y. The Molecular Characterization and Functional Analysis of Sexual Development Related Genes Sxl and Dmrt in the Prawn, Macrobrachium Rosenbergii. Hangzhou, Zhejiang: Zhejiang University. (2013).

[41] Ventura T Chandler JC Nguyen TV Hyde CJ Elizur A Fitzgibbon QP. Multi-Tissue Transcriptome Analysis Identifies Key Sexual Development-Related Genes of the Ornate Spiny Lobster (*Panulirus Ornatus*). Genes (Basel) (2020) 11:1–17. doi: 10.3390/genes11101150 PMC7600227 33003631

[42] Jiang J Yuan X Qiu Q Huang G Jiang Q Fu P. Comparative Transcriptome Analysis of Gonads for the Identification of Sex-Related Genes in Giant Freshwater Prawns (*Macrobrachium Rosenbergii*) Using RNA Sequencing. Genes (Basel) (2019) 10:1–18. doi: 10.3390/genes10121035 PMC6947849 31835875

[43] Matson CK Murphy MW Sarver AL Griswold MD Bardwell VJ Zarkower D. DMRT1 Prevents Female Reprogramming in the Postnatal Mammalian Testis. Nature (2011) 476:101–5. doi: 10.1038/nature10239 PMC3150961 21775990

[44] Farhadi A Fang S Zhang Y Cui W Fang H Ikhwanuddin M. The Significant Sex-Biased Expression Pattern of Sp-Wnt4 Provides Novel Insights Into the Ovarian Development of Mud Crab (*Scylla Paramamosain*). Int J Biol Macromol (2021) 183:490–501. doi: 10.1016/j.ijbiomac.2021.04.186 33957197

[45] Fan Z Zou Y Liang D Tan X Jiao S Wu Z. Roles of Forkhead Box Protein L2 (Foxl2) During Gonad Differentiation and Maintenance in a Fish, the Olive Flounder (*Paralichthys Olivaceus*). Reprod Fertil Dev (2019) 31:1742–52. doi: 10.1071/RD18233 31537253

[46] Gilbert LI. Halloween Genes Encode P450 Enzymes That Mediate Steroid Hormone Biosynthesis in Drosophila Melanogaster. Mol Cell Endocrinol (2004) 215:1–10. doi: 10.1016/j.mce.2003.11.003 15026169

[47] Lazar MA. Maturing of the Nuclear Receptor Family. J Clin Invest (2017) 127:1123–5. doi: 10.1172/JCI92949 PMC5373857 28368290

[48] Finger DS Whitehead KM Phipps DN Ables ET. Nuclear Receptors Linking Physiology and Germline Stem Cells in *Drosophila* . Vitam Horm (2021) 116:327–62. doi: 10.1016/bs.vh.2020.12.008.Nuclear PMC8063499 33752824

[49] Kiuchi T Koga H Kawamoto M Shoji K Sakai H Arai Y. A Single Female-Specific piRNA Is the Primary Determiner of Sex in the Silkworm. Nature (2014) 509:633–6. doi: 10.1038/nature13315 24828047

[50] Zhou LX Liu X Ye BQ Liu Y Tan SP Ma KY. Molecular Characterization of Ovary-Specific Gene Mrfem-1 and siRNA-Mediated Regulation on Targeting Mrfem-1 in the Giant Freshwater Prawn, Macrobrachium Rosenbergii. Gene (2020) 754:144891. doi: 10.1016/j.gene.2020.144891 32535048

[51] Ma KY Liu ZQ Lin JY Li JL Qiu GF. Molecular Characterization of a Novel Ovary-Specific Gene Fem-1 Homolog From the Oriental River Prawn, Macrobrachium Nipponense. Gene (2016) 575:244–52. doi: 10.1016/j.gene.2015.08.070 26367327

[52] Qiu GF Yamano K Unuma T. ). Cathepsin C Transcripts Are Differentially Expressed in the Final Stages of Oocyte Maturation in Kuruma Prawn Marsupenaeus Japonicus. Comp Biochem Physiol - B Biochem Mol Biol (2005) 140:171–81. doi: 10.1016/j.cbpc.2004.09.027 15649764

[53] Zhao W Chen L Zhang F Wu P Li E Qin J. Molecular Characterization of Cathepsin L cDNA and Its Expression During Oogenesis and Embryogenesis in the Oriental River Prawn Macrobrachium Nipponense (Palaemonidae). Genet Mol Res (2013) 12:5215–25. doi: 10.4238/2013.October.30.6 24301782

[54] Yang X Ikhwanuddin M Li X Lin F Wu Q Zhang Y. Comparative Transcriptome Analysis Providesinsights Into Differentially Expressed Genes and Long Non-Coding RNAs Between Ovary and Testis of the Mud Crab (*Scylla Paramamosain*). Mar Biotechnol (2018) 20:20–34. doi: 10.1007/s10126-017-9784-2 29152671

[55] Christians ES. Heat Shock Proteins and Maternal Contribution to Oogenesis and Early Embryogenesis. Adv Anat Embryol Cell Biol (2017) 222:1–27. doi: 10.1007/978-3-319-51409-3_1 28389748

[56] Jee B Dhar R Singh S Karmakar S. Heat Shock Proteins and Their Role in Pregnancy: Redefining the Function of “Old Rum in a New Bottle”. Front Cell Dev Biol (2021) 9:648463. doi: 10.3389/fcell.2021.648463 33996811 PMC8116900

[57] Nakkrasae L-I Damrongphol P. A Vasa-Like Gene in the Giant Freshwater Prawn, Macrobrachium rosenbergii. Mol Reprod Dev (2007) 1385:8–9. doi: 10.1002/mrd 17186538

[58] Park SY Jeong MS Han CW Yu HS Jang SB. Structural and Functional Insight Into Proliferating Cell Nuclear Antigen. J Microbiol Biotechnol (2016) 26:637–47. doi: 10.4014/jmb.1509.09051 26699741

[59] Xie Y Wang B Li F Jiang H Xiang J. Molecular Cloning and Characterization of Proliferating Cell Nuclear Antigen (PCNA) From Chinese Shrimp Fenneropenaeus Chinensis. Comp Biochem Physiol - B Biochem Mol Biol (2008) 151:225–9. doi: 10.1016/j.cbpb.2008.07.006 18678269

[60] Chen J Liu P Li Z Chen Y Qiu GF. The Cloning of the Cdk2 Transcript and the Localization of Its Expression During Gametogenesis in the Freshwater Giant Prawn, *Macrobrachium Rosenbergii* . Mol Biol Rep (2013) 40:4781–90. doi: 10.1007/s11033-013-2574-7 23653005

