# Supplementary material for: Full Functional Sex Reversal Achieved Through Silencing of MroDmrt11E Gene in Macrobrachium rosenbergii: Production of All-Male Monosex Freshwater Prawn
Source: Front Endocrinol (Lausanne). 2022 Mar 17;12:772498. doi: 10.3389/fendo.2021.772498 (PMC8970045; doi:10.3389/fendo.2021.772498)
Supplement: Supplementary file 1 [file DataSheet_1.docx]

Table 1S Significantly differential expression genes in four categories pathways of KEGG analysis

| **Gene name** | **Gene ID** | **MroDmrt11E**  **RNAi Readcount** | **GFP RNAi**  **Readcount** | **log2Foldchange** | **P value** | **Q value** | **Expression** |
| --- | --- | --- | --- | --- | --- | --- | --- |
| **Apoptosis** |  |  |  |  |  |  |  |
| **α-tubulin** | Cluster-19843.18842 | 110.63 | 42.99 | 1.36 | 4.90E-07 | 2.97E-05 | up |
| **α-tubulin** | Cluster-19843.18843 | 1050.86 | 406.83 | 1.37 | 1.35E-54 | 1.62E-51 | up |
| **α-tubulin** | Cluster-19843.9908 | 112.99 | 44.36 | 1.35 | 4.77E-07 | 2.90E-05 | up |
| **α-tubulin** | Cluster-19843.22263 | 2754.76 | 1030.48 | 1.42 | 6.63E-149 | 2.78E-145 | up |
| **Actin** | Cluster-19843.26904 | 76.81 | 28.27 | 1.44 | 1.10E-05 | 4.90E-04 | up |
| **Actin** | Cluster-19843.28340 | 108.49 | 20.25 | 2.42 | 1.39E-14 | 2.48E-12 | up |
| **Cathepsin** | Cluster-19843.27411 | 418.37 | 163.43 | 1.36 | 2.13E-22 | 7.24E-20 | up |
| **Cathepsin** | Cluster-19843.22524 | 4110.23 | 1594.17 | 1.37 | 1.19E-207 | 9.99E-204 | up |
| **Cathepsin** | Cluster-19843.21212 | 878.83 | 377.00 | 1.22 | 7.89E-38 | 5.44E-35 | up |
| **PI3K** | Cluster-19843.11089 | 68.47 | 24.25 | 1.50 | 1.83E-05 | 7.59E-04 | up |
| **Ras** | Cluster-19843.10363 | 266.40 | 125.05 | 1.09 | 1.80E-10 | 1.97E-08 | up |
| **eiF2α** | Cluster-19843.21660 | 91.80 | 240.84 | -1.39 | 9.94E-20 | 2.73E-17 | down |
| **Regulation of actin cytoskeleton** | |  |  |  |  |  |  |
| **IQGAP** | Cluster-19843.21583 | 148.23 | 53.11 | 1.48 | 4.21E-10 | 4.39E-08 | up |
| **Actin** | Cluster-19843.26904 | 76.81 | 28.27 | 1.44 | 1.10E-05 | 4.90E-04 | up |
| **Actin** | Cluster-19843.28340 | 108.49 | 20.25 | 2.42 | 1.39E-14 | 2.48E-12 | up |
| **Rac** | Cluster-19843.20363 | 72.98 | 29.99 | 1.28 | 1.08E-04 | 3.60E-03 | up |
| **Rac** | Cluster-19843.10591 | 110.58 | 52.29 | 1.08 | 4.68E-05 | 1.73E-03 | up |
| **HSPC300** | Cluster-19843.11023 | 50.85 | 107.21 | -1.08 | 3.86E-07 | 2.41E-05 | down |
| **Protein processing in endoplasmic reticulum** | | |  |  |  |  |  |
| **Sec62/63** | Cluster-19843.29293 | 168.27 | 75.15 | 1.16 | 7.48E-08 | 5.24E-06 | up |
| **Sec61** | Cluster-19843.20025 | 102.18 | 224.79 | -1.14 | 2.11E-14 | 3.70E-12 | down |
| **Hsp70** | Cluster-19843.20831 | 493.83 | 231.58 | 1.09 | 3.49E-18 | 8.52E-16 | up |
| **Hsp90** | Cluster-19843.20012 | 165.28 | 56.29 | 1.55 | 6.62E-12 | 8.62E-10 | up |
| **Hsp90** | Cluster-19843.21962 | 178.80 | 88.67 | 1.01 | 1.17E-06 | 6.49E-05 | up |
| **Hsp90** | Cluster-19843.25476 | 1479.40 | 647.20 | 1.19 | 5.86E-60 | 8.67E-57 | up |
| **Hsp90** | Cluster-19843.17033 | 170.42 | 65.03 | 1.39 | 2.20E-10 | 2.38E-08 | up |
| **P97** | Cluster-19843.18022 | 414.19 | 184.72 | 1.16 | 2.83E-17 | 6.38E-15 | up |
| **UbcH5** | Cluster-19843.21815 | 489.46 | 234.66 | 1.06 | 3.91E-17 | 8.67E-15 | up |
| **Ubc6/7** | Cluster-19843.21559 | 45.40 | 99.23 | -1.13 | 4.44E-07 | 2.72E-05 | down |
| **GlcII** | Cluster-19843.26272 | 26.72 | 65.89 | -1.30 | 5.50E-06 | 2.66E-04 | down |
| **OS9** | Cluster-19843.12255 | 35.83 | 72.11 | -1.01 | 6.98E-05 | 2.44E-03 | down |
| **TRAM** | Cluster-19843.18883 | 100.15 | 258.84 | -1.37 | 1.12E-20 | 3.31E-18 | down |
| **Skp1** | Cluster-19843.21006 | 414.54 | 904.37 | -1.13 | 2.60E-52 | 2.91E-49 | down |
| **Oocyte meiosis** | |  |  |  |  |  |  |
| **CPEB** | Cluster-19843.18308 | 704.49 | 344.06 | 1.03 | 6.63E-23 | 2.33E-20 | up |
| **Rec8** | Cluster-19843.21750 | 880.36 | 387.44 | 1.18 | 6.33E-36 | 4.03E-33 | up |
| **Calm** | Cluster-19843.24202 | 1319.54 | 436.27 | 1.60 | 1.37E-87 | 3.45E-84 | up |
| **CaN** | Cluster-19843.21548 | 76.11 | 32.21 | 1.24 | 1.24E-04 | 4.06E-03 | up |
| **CycE** | Cluster-19843.23974 | 222.12 | 90.75 | 1.29 | 1.09E-11 | 1.38E-09 | up |
| **Cdk2** | Cluster-19843.21750 | 880.36 | 387.44 | 1.18 | 6.33E-36 | 4.03E-33 | up |
| **SFC** | Cluster-19843.21006 | 414.54 | 904.37 | -1.13 | 2.60E-52 | 2.91E-49 | down |
